# Comparison of Ginsenoside Content and In Vitro Biological Activity of Extracts Derived from Hairy Root Cultures and Field-Cultivated Roots of *Panax quinquefolium*

**DOI:** 10.3390/molecules31122117

**Published:** 2026-06-16

**Authors:** Grażyna Szymańska, Weronika Gonciarz, Patrycja Jaroniek, Angelika Szymańska, Ewa Kochan

**Affiliations:** 1 Department of Pharmaceutical Biotechnology, Medical University of Lodz, Muszyńskiego 1, 90-151 Lodz, Poland; angelika.gabrysiak@umed.lodz.pl; 2 Department of Immunology and Infectious Biology, Institute of Microbiology, Biotechnology and Immunology, Faculty of Biology and Environmental Protection, University of Lodz, Banacha 12/16, 90-237 Lodz, Poland; weronika.gonciarz@uni.lodz.pl (W.G.);; 3 Bio-Med-Chem Doctoral School of the University of Lodz and Lodz Institutes of the Polish Academy of Sciences, University of Lodz, Matejki 21/23, 90-237 Lodz, Poland

**Keywords:** *Panax quinquefolium*, hairy root cultures, anti-inflammatory, antibacterial, cytotoxic properties

## Abstract

Field-cultivated roots of *Panax quinquefolium* represent the natural source of biologically active compounds, e.g., ginsenosides, while transformed roots provide a controlled alternative for their production. Ginsenoside levels from both the sources were determined with the use of the HPLC method. The extracts were tested for antimicrobial activity using the MIC and MBC/MFC methods, as well as for cytotoxic activity on the AGS (gastric cancer) cell line, Hs68 (human fibroblasts), and L929 (mouse fibroblasts) lines using the MTT assay. Additionally, the lack of pro-inflammatory activity of the plant materials was assessed using a monocyte activation test. The tested *P. quinquefolium* roots differed quantitatively and qualitatively in their ginsenoside profiles, and the highest amount was recorded in the transformed roots (204.62 ± 5.56 mg/g extract ± SE). The extracts exhibited the strongest antimicrobial activity against the *Escherichia coli* strain. Low activity of the tested extracts was observed against *Candida* species. In the tested cell lines (AGS, Hs68, L929), a dose-dependent decrease in cell viability was observed, with the field root extract exhibiting the highest cytotoxic activity in the concentration range of 2.5–10 mg/mL. All tested extracts proved to be safe and did not stimulate a pro-inflammatory response.

## 1. Introduction

The *Panax* genus includes many species of plants which demonstrate a wide range of pharmacological properties and which have been used in natural medicine for many generations [1,2]. These well-known plants belonging to the *Araliaceae* family are commonly found in as many as 64 countries and are cultivated mainly in Asia, in South Korea and China [3]. *Panax quinquefolium* (American ginseng) and *Panax ginseng* are reported to be most commonly used in medicine and also best studied [4,5,6,7]. Monographs of these species can be found in the Chinese, American and European pharmacopeias [8]. The ginseng plant has been in the center of scientific interest and subject of numerous extensive studies due to its pharmacological properties, mechanisms of action, and main active compounds. Since there is a need to systematize and summarize findings from multiple studies, this species is also frequently described in review papers and meta-analyses.

Ginsenosides are considered to be the main compounds responsible for the pharmacological and chemical effects of ginseng [9]. With regard to their structure, they can be divided into primary ginsenosides (Rg1, Re, Rd, etc.) and secondary ginsenosides (Rg5, Rk1, Rg3, etc.). The latter are formed by hydrolysis of primary ginsenosides. Such hydrolysis can occur by, for example, steaming, acid/base treatment or microbiological metabolic transformation. Ginsenosides, which are chemically glycosylated triterpenoids, can be classified into four main types on the basis of their structure: protopanaxatriol (PPT), which includes ginsenosides such as Rh1, Rh2, Rg1, Rg2, Re, Rf, and protopanaxadiol (PPD), including Rb1, Rb2, Rc, Rd, Rg3, and oleanane (OA), which includes Ro and, finally, octillol (OCT) [10,11].

Due to its pharmacological properties, the ginseng plant is particularly popular among researchers. In recent years, the number of reviews and innovative studies documenting its beneficial effects has grown. Although these studies demonstrate pharmacological effects of various ginseng species, researchers most often conduct their experiments on one species, *Panax ginseng*. These studies provide evidence for its anti-inflammatory, antimicrobial, antioxidant, immunomodulatory, neuroprotective, cardioprotective, and adaptogenic effects. These properties are primarily attributed to the presence of ginsenosides. However, other biologically active components are also found in this plant [7,12,13,14,15].

Despite significant progress in oncology, cancer is still one of leading causes of global mortality. Current therapeutic strategies are often associated with very difficult treatment courses and numerous burdensome side effects and toxicities for the patient. Therefore, natural compounds with anticancer potential are constantly being searched for. Such compounds are believed to demonstrate fewer side effects and a better safety profile compared to conventional chemotherapeutics, which might open new avenues for cancer treatment. Numerous studies conducted in recent years have confirmed that ginseng has the potential to inhibit the development of skin, breast, lung, liver, colon, gastric, ovarian, kidney cancers and leukemia. For example study conducted on lung cancer cells showed that ginsenoside Rb1 inhibited the proliferation and migration and induced apoptosis of lung cancer cells by regulating the mitochondrial apoptosis pathway, which led to anticancer activity [16]. These data indicate that *Panax ginseng* is highly biologically effective in limiting the growth of a variety of cancer cells and thus can potentially serve as a natural adjunct to anticancer therapy.

Inflammation is the body’s fundamental biological, protective response to infection or injury.

However, dysregulated or chronic inflammation contributes to the development of numerous pathological conditions, including cardiovascular disease, autoimmune diseases, neurodegenerative diseases, metabolic syndromes, and even some cancers. Therefore, the ability to control or regulate the immune response may prove crucial in preventing complications stemming from chronic inflammation. Ginseng demonstrates its valuable activity in this regard, and research conducted over the years suggests that extracts from this plant may be helpful in regulating inflammation by reducing the activity of inflammatory mediators and limiting oxidative stress [17,18].

The growing incidence of antimicrobial resistance poses a serious global health threat. This situation fuels the need to search for new antimicrobial agents. Plant-based substances with such properties appear to be an ideal alternative, as they may prove safer and may have fewer side effects compared to other antimicrobial agents, and ginseng, in addition to its anticancer and anti-inflammatory effects, exhibits antimicrobial activity against a range of pathogens. This aspect has been less extensively studied than other pharmacological properties. However, a growing body of evidence suggests that ginsenosides may interfere with microbial growth. These findings highlight the potential of ginseng-derived compounds as natural antimicrobial agents, particularly in the context of increasing antimicrobial resistance.

Much of the research on pharmacological properties of ginseng is based on plant material grown under field conditions. However, the chemical composition, content, and quality of ginsenosides are strongly influenced by environmental factors such as light intensity, temperature, soil composition, cultivation methods, and geographic origin, which can lead to significant variability in phytochemical profiles.

To overcome these limitations, in vitro culture systems are being developed for various *Panax* species, including *Panax ginseng*, *Panax quinquefolium*, and *Panax notoginseng*. These systems include callus cultures, adventitious roots, and transformed roots. They are primarily used as alternative sources for biomass production and ginsenoside biosynthesis.

To date, research on in vitro cultures of *Panax* species has focused primarily on optimization of growth conditions, increase in biomass accumulation, and quantitative analysis of ginsenoside content. Relatively few studies have examined the biological activity of extracts derived from in vitro cultured material. In vitro systems offer several significant advantages over conventional field cultivation. They provide strictly controlled and repeatable growth conditions, independent of environmental and seasonal variations, and reduce the risk of pathogen contamination. Furthermore, in vitro cultures enable the use of elicitors and precise manipulation of culture parameters to enhance the production of specific secondary metabolites. Importantly, field cultivation of ginseng is a time-consuming and labor-intensive process, in which several years are often needed to obtain mature roots. Therefore, in vitro cultures represent a promising alternative source of biomass for pharmaceutical and biotechnological research. However, despite significant progress in the development of in vitro cultivation methods for *Panax* species, further research is necessary to comprehensively assess biological properties of extracts obtained with the use of these systems and compare their activity with that of material grown in the field [19,20,21].

The aim of this investigation was a comparative analysis of ginsenoside content in field-cultivated roots and hairy root cultures of *P. quinquefolium*. In addition to phytochemical characterization, the extracts obtained were assessed for biological activity.

Their antimicrobial activity was evaluated against selected reference strains of Gram-positive and Gram-negative bacteria, as well as *Candida* strains.

While reports on the anticancer effects of extracts from various ginseng species can be found in the literature, to the best of our knowledge, this is the first study to evaluate the cytotoxic effects of extracts of *P. quinquefolium* hairy root cultures on three cell lines, including normal fibroblast lines (L929 and Hs68) and a human gastric adenocarcinoma cell line (AGS). Furthermore, the extracts derived from American ginseng transformed roots have not been previously evaluated for their safety and potential to induce inflammatory reactions.

## 2. Results

### 2.1. Ginsenoside Content in Extracts of P. quinquefolium

**Figure 1:**
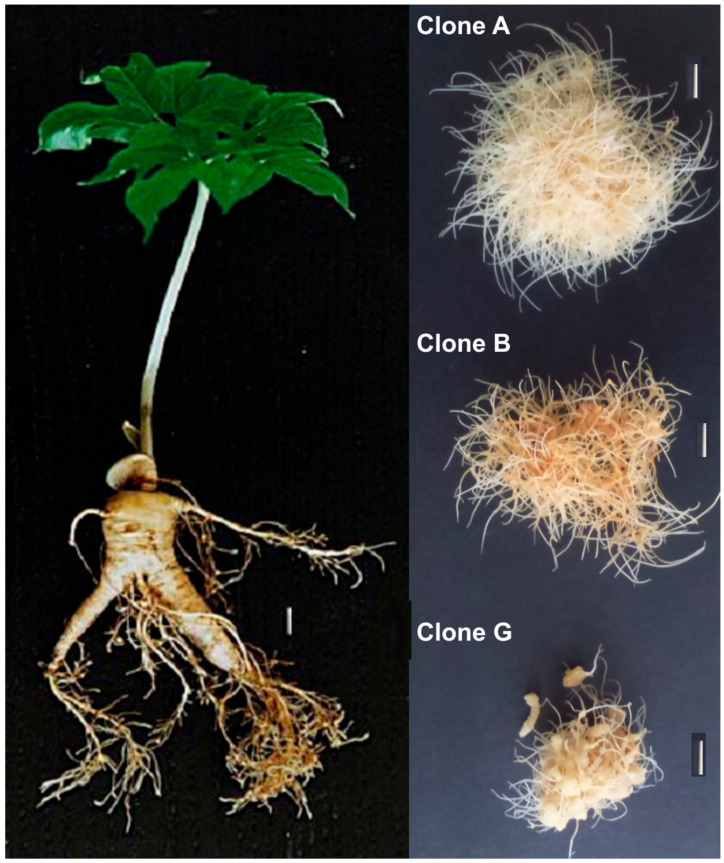
*P. quinquefolium* roots used in research: 3-year-old field-cultivated roots and three clones of hairy root cultures (A, B, G).

**Figure 2:**
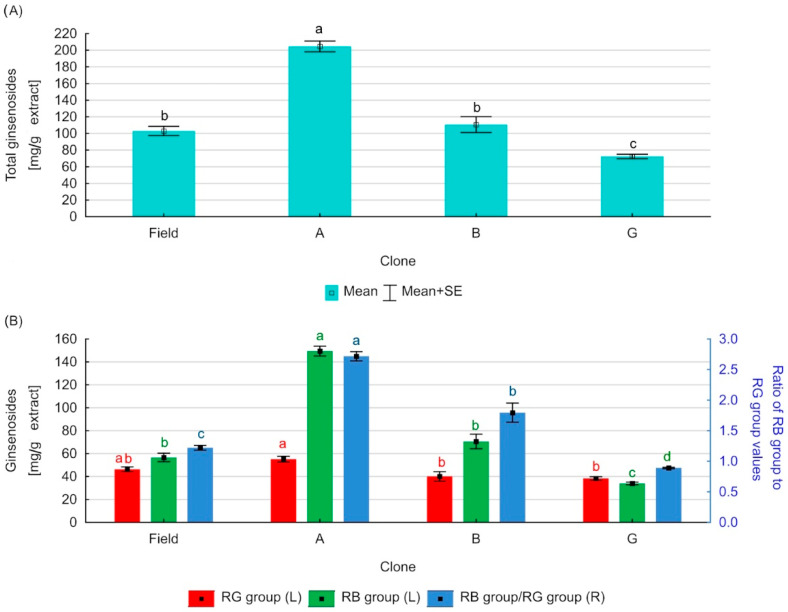
Saponin levels ((**A**)—total saponins; (**B**)—RB group saponins; RG group saponins and the relationship between ginsenosides from the RB group and the RG group) in field-cultivated roots and hairy root cultures of *P. quinquefolium*. Experiments were performed in triplicate by three independent experimenters. Data are presented as mean values ± SE. Data were analyzed using the Kruskal–Wallis test. The same lowercase letters indicate no statistically significant differences.

Eight ginsenosides (Rb1, Rb2, Rb3, Rc, Rd, Re, Rg1, Rg2) were quantified in extracts derived from four groups of studied plant material: field-cultivated roots and clones A, B and G of hairy root cultures of *P. quinquefolium* (Figure 1). In field-cultivated roots, the content of all studied saponins was 102.99 ± 5.56 mg/g of extract ± SE (Figure 2A). The obtained results indicated substantial differences among the clones of transformed root cultures. The highest level of ginsenosides (204.62 ± 5.56 mg/g of extract ± SE) was observed in clone A rather than in clones B or G. That level was twice as high as in clone B (110.64 ± 9.51 mg/g of extract ± SE) and field-cultivated roots and almost three times higher than in clone G (72.36 ± 2.67 mg/g of extract ± SE).

**Figure 3:**
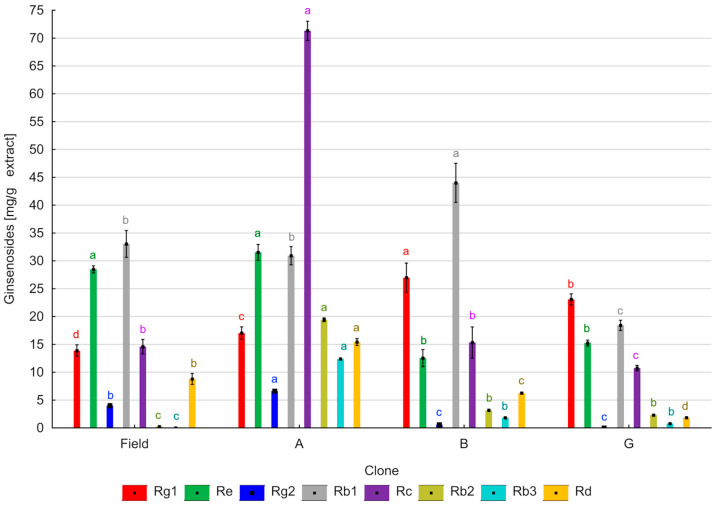
Levels of individual ginsenosides in field-cultivated roots and hairy root cultures of *P. quinquefolium*. Experiments were performed in triplicate by three independent experimenters. Data are presented as mean values ± SE. Data were analyzed using the Kruskal–Wallis test. The same lowercase letters indicate no statistically significant difference.

The content of protopanaxadiol derivatives (the total of Rb1, Rb2, Rc and Rd, group RB) was significantly higher in clone A of hairy root cultures (149.44 ± 4.26 mg/g of extract ± SE compared to the other tested plant material), while the lowest saponin level of the RB group was noted in clone G (34.05 ± 1.17 mg/g of extract ± SE). A similar tendency was observed for protopanaxatriol derivatives (the total of Re, Rg1 and Rg2, group RG) (Figure 2B). On the basis of the level of protopanaxadiol and protopanaxatriol derivatives, the relationship between RB group ginsenosides and RG group saponins was also determined. The value of the RB group/RG group parameter ranged between 0.9 and 2.7 depending on the type of plant material (Figure 2B). The highest level of this parameter was found in clone A of hairy root cultures, and it reached the value 2.7, whereas the lowest one was noted for clone G of hairy roots (0.9). Quantitative analysis revealed that the content of individual ginseng saponins was significantly different in the tested plant material (Figure 3). Hairy roots of clone A and B produced all examined metabolites. Saponin Rg2 was absent in clone G. Field-cultivated roots did not produce ginsenosides Rb3. In turn, the saponins Rb1, Rc and Rg1 were quantitatively dominant depending on the studied samples. The maximum content of ginsenosides Rb1 (almost 44 ± 3.51 mg/g of extract ± SE was found in extracts obtained from clone B of hairy roots). That metabolite also predominated in field-cultivated roots (almost 33.03 ± 2.4 mg/g of extract ± SE). The highest content of Rc (71.32 ±1.72 mg/g of extract ± SE) was noted in clone A. Rg1 (23.08 ± 1.0 mg/g of extract ± SE) was quantitatively dominant in clone G.

**Figure 4:**
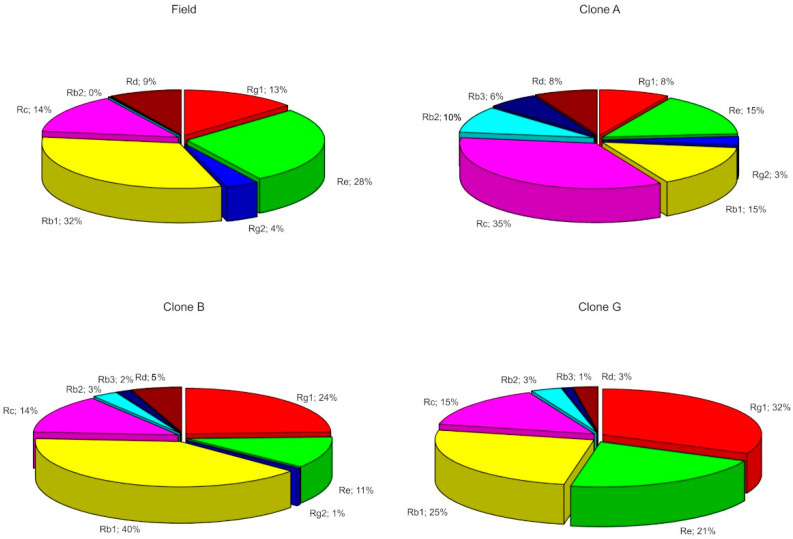
The percentage value of individual ginsenosides in relation to all studied compounds in field-cultivated roots and hairy root cultures of *P. quinquefolium*.

The proportion of individual ginsenosides in relation to all tested compounds was also established in three clones of hairy roots and in the field-cultivated roots (Figure 4). A comparison of the profiles of individual compounds in field-cultivated roots and clones of hairy roots revealed that they are completely different. The diagram presenting the order of percentage values of individual saponins is as follows:

Field root: Rb1 > Re > Rc > Rg1 > Rd > Rg2 > Rb2;

Clone A: Rc > Re~Rb1 > Rb2 > Rd~Rg1 > Rb3 > Rg2;

Clone B: Rb1 > Rg1 > Rc > Re > Rd > Rb2 > Rb3 > Rg2;

Clone G: Rg1 > Rb1 > Re > Rc > Rd~Rb2 > Rb3.

### 2.2. Biological Properties of Extracts of P. quinquefolium

#### 2.2.1. Antimicrobial Properties of Studied Plant Material

Obtained *P. quinquefolium* extracts were tested for their antibacterial and antifungal activity. Results revealed that A, B, and G clones of hairy roots, as well as field root extracts, exhibited weak antibacterial and antifungal activity in comparison to standard antibiotics (gentamicin and amphotericin B).

**Figure 5:**
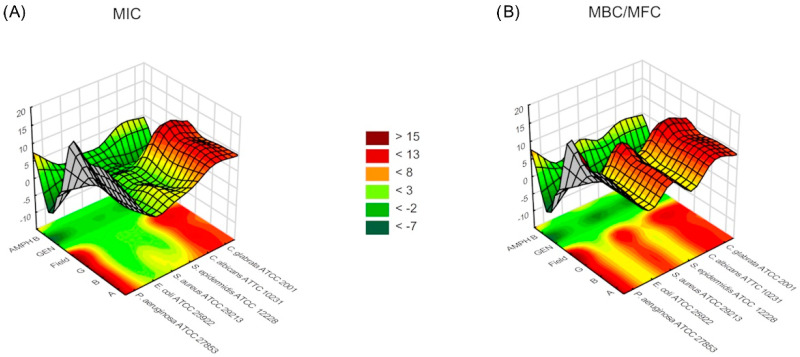
Antimicrobial activity of in vitro and traditionally cultivated root extracts against studied microorganisms: (**A**)-MIC value; (**B**)-MBC/MFC value. Experiments were performed in triplicate by three independent experimenters.

Figure 5 presents two three-dimensional surface graphs comparing the antimicrobial activity of the tested extracts (expressed as MIC and MBC/MFC values) against several selected strains of microorganisms. Table S1 in the Supplementary File presents complete MIC, MBC, and MFC data. Plot A represents the lowest concentration required to inhibit visible growth of microorganisms, whereas the color scale indicates MIC values, where the green color represents lower values (stronger inhibitory activity) and the red one represents higher values (weaker activity). The strongest inhibitory properties, with an MIC value of 1.25 mg/mL, were observed for extracts from clone B and G of hairy roots and field-cultivated roots against *Escherichia coli*. By contrast, results concerning another Gram-negative bacterium—*Pseudomonas aeruginosa*—showed that the MIC value was significantly higher, i.e., above 10 mg/mL for all tested extracts.

Different observations were made for the tested strains of Gram-positive bacteria: *S. epidermidis* and *S. aureus*. The lowest MIC value (2.5 mg/mL) was recorded for extracts derived from clone G of transformed roots and field-cultivated roots. However, the determined MIC value (2.5 mg/mL) was twice as high as that observed for *E. coli*. The *S. epidermidis* strain was also sensitive to the extract obtained from clone B, which inhibited its growth at a concentration of 2.5 mg/mL. Furthermore, the obtained results revealed that the antifungal activity of *P. quinquefolium* extracts against *Candida* species (MIC value of 10 mg/mL) was low. Plot B (MBC/MFC—Minimum Bactericidal/Fungicidal Concentration) demonstrates the concentration of tested extracts which was required to kill the microorganisms. The layout is similar to the MIC plot, with the same strains and plant material samples. Similarly, green areas indicate stronger killing activity (lower MBC/MFC), while red/orange areas indicate weaker effects. The MBC/MFC values were generally higher than the MICs and ranged from 2.5 to over 10, depending on the type of extract and the microorganism.

#### 2.2.2. Cytotoxic Activity of Studied Plant Material

**Figure 6:**
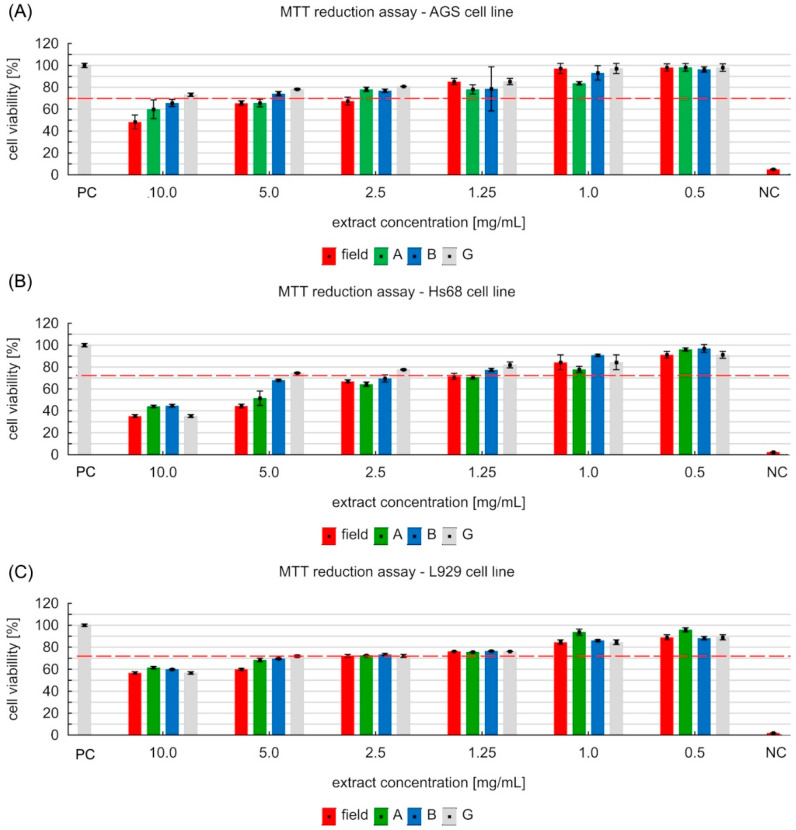
The effect of extracts derived from field-cultivated roots and hairy root clones (A, B, and G) of *Panax quinquefolium* on the cell viability of three cell lines: AGS (gastric cancer, (**A**)), Hs68 (human fibroblasts, (**B**)), and L929 (mouse fibroblasts, (**C**)) with the use of MTT reduction assay. Experiments were performed in triplicate by three independent experimenters. Data are presented as mean values ± SD. Data were analyzed using the Mann–Whitney U test. The dashed red line (~70%) indicating the cytotoxicity threshold according to ISO standards.

To evaluate the cytotoxic activity of extracts derived from field-cultivated roots and hairy root clones (A, B, and G), cell viability in three cell lines: AGS (gastric cancer, Figure 6A), Hs68 (human fibroblasts, Figure 6B), and L929 (mouse fibroblasts, Figure 6C) was assessed using the MTT reduction assay. Cell viability is expressed as a percentage value relative to the positive control (PC), with the dashed red line (~70%) indicating the cytotoxicity threshold according to ISO standards.

In all tested cell lines, a dose-dependent decrease in cell viability was observed (Figure 6). In AGS cells, the field root extract showed the highest cytotoxic activity in the concentration range of 2.5–10 mg/mL (37–52 ± 1% of dead cells), followed by the A clone extract (concentration: 5–10 mg/mL; 35–40 ± 1.7% of dead cells). The B clone extract showed activity only in the highest concentration range (10 mg/mL; 34 ± 2% of dead cells). The G clone extract showed no cytotoxic activity against the AGS line in the entire range of the tested concentrations (less than 30% of dead cells) (Figure 6A). According to the ISO norm the A, B, and G clones of hairy root and field root extract showed no cytotoxic activity against mouse L929 and human Hs68 fibroblasts within the concentration range of 0.5–2.5 mg/mL (5–28 ± 1.8% of dead cells). The weakest effect was noted for extracts derived from the G clone (10–29 ± 1.5% of dead cells in the concentration range of 0.5–5 mg/mL) (Figure 6B,C). The L929 fibroblast cell line was the least sensitive to all tested samples, as none of the treatments reduced cell viability below 50% within the tested concentration range. In contrast, Hs68 fibroblasts showed greater susceptibility, particularly to the field sample (IC_50_ ≈ 4.4), followed by sample A (IC_50_ ≈ 6.0). The AGS gastric cancer cell line exhibited relatively high resistance, with only the field sample achieving an IC_50_ within the tested concentration range (IC_50_ ≈ 9.5).

Overall, the field sample demonstrated the strongest cytotoxic activity among all tested preparations. However, since the IC_50_ values obtained for normal fibroblasts (Hs68) were lower than those observed for AGS cancer cells, the results do not indicate selective anticancer activity under the experimental conditions. Instead, the cytotoxic effect appears to be more pronounced towards normal fibroblast cells than towards gastric cancer cells.

##### Classical Selectivity Index (SI)

The classical selectivity index (SI), defined as the ratio of IC_50_ values in normal cells to cancer cells, was used to assess the differential cytotoxicity of the tested samples. Using Hs68 fibroblasts as the representative normal human cell line and AGS gastric cancer cells as the malignant model, both sample A and sample B exhibited SI values below 1 (SI_A < 0.60; SI_B < 0.89) (Table 1).

An SI value below 1 indicates that the tested compounds are not selectively cytotoxic toward cancer cells but instead show equal or greater toxicity to normal cells compared to cancer cells. In particular, both samples displayed measurable activity in normal fibroblasts (Hs68 IC_50_ ~6.0–8.9), while showing weak or no cytotoxicity in AGS cells (IC_50_ > 10), further confirming the lack of tumor selectivity.

The cross-species selectivity analysis comparing murine L929 fibroblasts and human AGS gastric cancer cells reveals that all evaluated samples demonstrate no clear differential cytotoxicity between normal and cancer cells. For samples A, B, and G, both L929 and AGS cell lines showed IC_50_ values greater than 10 µg/mL, resulting in an approximate selectivity index close to unity (SI ≈ 1), which suggests a lack of preferential toxicity towards either cancer or normal cells.

This pattern indicates a non-selective cytotoxic profile, where the tested compounds do not effectively discriminate between malignant and non-malignant cellular systems across species. Although L929 cells (mouse fibroblasts) are often more sensitive than human fibroblasts, no such differential sensitivity was observed here, reinforcing the conclusion of limited therapeutic selectivity.

##### Relationship Between Antimicrobial Activity and Cytotoxicity

Comparison of antimicrobial and cytotoxic properties indicates that the strongest antimicrobial activity was generally observed for the G and field samples, particularly against *E. coli* and staphylococci, where MIC values ranged from 1.25 to 2.5 mg/mL.

The field sample exhibited the broadest biological activity profile, showing both potent antibacterial effects and the lowest IC_50_ values in mammalian cells. However, its cytotoxicity toward normal fibroblasts (Hs68; IC_50_ ≈ 4.4 mg/mL) was observed at concentrations only approximately two- to four-fold higher than the antibacterial MIC values (1.25–2.5 mg/mL), suggesting a relatively narrow therapeutic window.

Sample G appears particularly promising because it displayed antimicrobial activity comparable to field values (MIC = 1.25–2.5 mg/mL against susceptible bacteria) while exhibiting substantially lower cytotoxicity (IC_50_ > 10 mg/mL in all tested cell lines). This indicates a more favorable selectivity profile.

#### 2.2.3. Activation of the Transcription Factor NF-kB

*P. quinquefolium* root extracts, obtained in conventional and in vitro cultivation, were also tested for anti-inflammatory properties.

**Figure 7:**
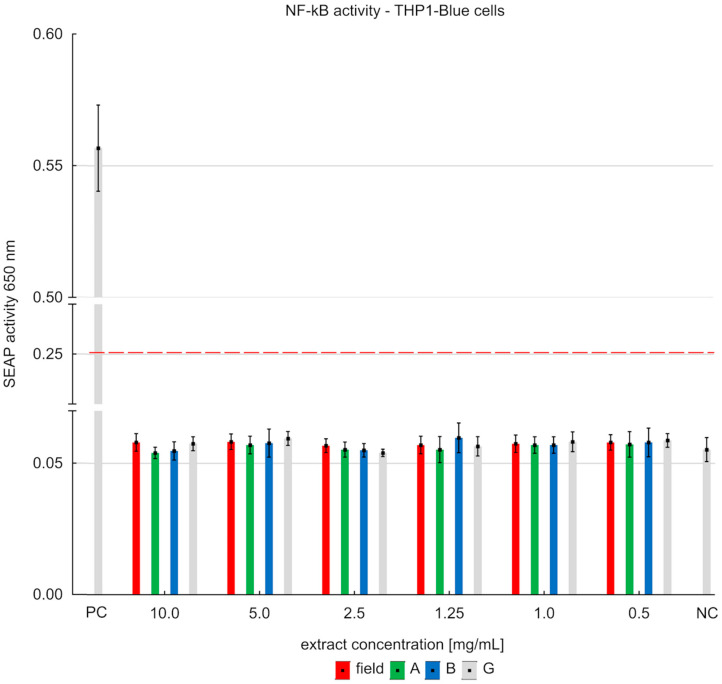
NF-κB activation in THP1-Blue™ monocytes expressed as SEAP activity after treatment with extracts derived from field-cultivated roots and hairy root clones (A, B, and G) of *P. quinquefolium* at different concentrations. Experiments were performed in triplicate by three independent experimenters. Data are presented as mean values ± SD. Data were analyzed using the Mann–Whitney U test. The dashed red line (~0.25) indication the threshold activation.

For this purpose, an experimental model was used in which LPS-stimulated THP-1 x Blue monocytes responded by generating high SEAP levels, indicating activation of the nuclear transcription factor NF-κB. The positive control (PC) induced strong NF-κB activation, reaching the level of approximately 0.55, which is significantly above the activation threshold indicated by the dashed red line (~0.25). Field root extracts, as well as extracts from hairy root clones A, B, and G, showed very low levels of SEAP activity at all tested concentrations (0.5–10 mg/mL). The obtained values were similar to the negative control (NC) and remained below the activation threshold, which confirmed the immunosafety of the tested extracts and indicated the absence of NF-κB activation (Figure 7).

## 3. Discussion

The available literature focuses primarily on characterizing biological properties of the most well-known ginseng, *Panax ginseng*. In these reports, the extracts derived mainly from traditional field-cultivated roots were tested for their antimicrobial, anticancer, antioxidant and anti-inflammatory properties [18,22].

In this paper, the content of ginsenosides and antimicrobial and cytotoxic properties were evaluated in extracts of *P. quinquefolium* roots, obtained both from field-cultivated roots and hairy root cultures. In addition, the absence of NF-κB activation was also investigated.

The obtained results showed that *P. quinquefolium* roots differed significantly in their ginsenoside profiles, both quantitatively and qualitatively. The total saponin amount, recorded in traditionally cultivated roots, was 2–4.8-fold higher than in field-cultivated *P. ginseng* roots [23], but at the same time, it was two times lower than the content of ginsenosides in clone A of hairy root cultures. These results indicated that hairy root cultures may exhibit enhanced biosynthetic capacity, which may be due to stable genetic transformation and controlled growth conditions favoring the accumulation of secondary metabolites.

Our findings also revealed that ginsenoside level in clone A of hairy roots was significantly higher not only than the saponin content in field-cultivated roots but also higher than that in other transformed clones. Such variability between clones is a well-known phenomenon and it is attributed to differences in T-DNA integration sites, gene expression levels, and regulation of metabolic pathways following *Agrobacterium rhizogenes* transformation [24,25,26].

The analysis of protopanaxadiol (RB group) and protopanaxatriol (RG group) derivatives revealed that clone A accumulated the highest levels of both fractions, while clone G showed the lowest levels. Furthermore, the ratio of RB group/RG group saponins varied considerably among the samples tested, ranging from 0.9 to 2.7.

In the ginseng plant grown in different regions, the ratio between protopanaxadiol derivatives and protopanaxatriol derivatives ranged from 1.36 even up to 20.06, depending on the type of ginseng samples [27] and steaming time after harvest [23].

**Figure 8:**
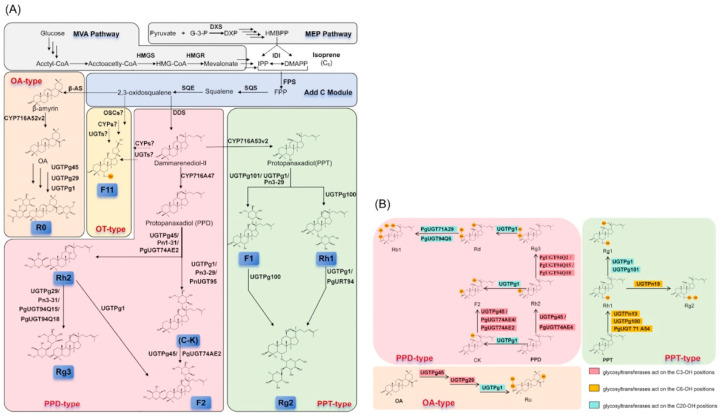
Biosynthetic pathway of ginsenosides. (**A**) G-3-P, Glyceraldehyde 3-phosphate; DXP, 1-Deoxy-D xylulose 5-phosphate; HMBPP, 4-Hydroxy-3-methylbut-2-enyl diphosphate; HMG-CoA, 3-Hydroxy 3-methylglutaryl-coenzyme A; IPP, isopentanyl pyrophosphate; DMAPP, 3,3-dimethylallyl diphos phate; FPP, farnesyl pyrophosphate; FPS, farnesyl pyrophosphate synthase; SQS, squalene synthase; SQE, squalene epoxidase; DDS, dammarenediol synthase; CYP716A47, CYP716A53v2, and CYP716A52v2 are all Cytochrome P450 oxidases enzymes; PPD, protopanaxadiol; PPT, protopanax atriol; β-AS, β-amyrin synthase; OA, oleanolic acid. R0, F11, Rh2, Rg3, C-K, F2, F1, Rh1, and Rg2 are all commonly used abbreviations for various ginsenosides. The background colors indicate different biosynthetic pathways: gray for the precursor synthesis MEP and MVA pathways; blue for the carbon chain elongation process; orange for the biosynthesis of OA-type ginsenosides; light yellow for OT-type, pink for PPD-type, and green for PPT-type ginsenosides. (**B**) Glycosyltransferases: types and action sites [29].

Importantly, this parameter shows which branch of ginsenoside biosynthesis is preferred (Figure 8). Elevated RB/RG ratios, like those observed in clone A, reveal that protopanaxadiol derivatives are predominant, whereas lower ratios suggest an increase in the number of protopanaxatriol-type compounds. Thus, the relationship between the RB group and RG group saponins also reflects the trend of modifications of individual ginsenoside structures in the final stages of their biosynthesis. Furthermore, Tian et al. found that the ratio between protopanaxadiol-type ginsenosides and protopanaxatriol-type ginsenosides (PPD/PPT) was characteristic of the ginseng species and was as follows: 0.69, 1.11, and 2.19 for *Panax notoginseng*, *P. ginseng*, and *P. quinquefolium* respectively. In addition, it was consistent with the expression levels of CYP716A53, the enzyme catalyzing the 6-hydroxylation of PPD to produce PPT [28].

Furthermore, qualitative and quantitative differences in individual ginsenoside composition were noted in the studied extracts. For example, the absence of Rg2 in clone G and Rb3 in field-cultivated roots was observed. Similar observations were reported in other studies, which proved that the presence or absence of individual metabolites depended on both genetic background (i.e., ginseng species) as well as growth and culture conditions [30,31]. On the other hand, in our studies clear dominance of some ginsenosides—such as Rb1 in clone B, Rc in clone A, and Rg1 in clone G—was observed. This effect may arise from variations in the regulation of glycosylation processes, which play a key role in determining the structural diversity and biological functions of ginsenosides. Recent studies have revealed that the changes in the levels of individual saponins may be linked to variations in the expression of genes coding enzymes, such as cytochrome P450 as well as glycosyltransferases, which are involved in the structural diversification of ginsenosides [29,32]. Our results indicate that it is highly probable that the expression of these genes is regulated differently, both in individual clones of transformed roots and field-cultivated roots.

Obtained extracts of *P. quinquefolium* were tested for antimicrobial, cytotoxic and absence of NF-κB activation properties.

Antibiotics are widely used worldwide to treat infectious diseases. However, they are overused and misused in the general population, which results in increased antibiotic resistance rates among several microorganisms [33,34]. Consequently, efforts have been made to counteract the threat of antibiotic resistance and simultaneously to explore alternative sources of antimicrobial agents, such as medicinal plants [35]. American ginseng, a species of ginseng originating from North America is an example of such a plant. Investigating antibacterial properties of ginseng extracts is important because they may provide a natural source of bioactive compounds that will be capable of combating microbial infections, thereby counteracting rising antibiotic resistance. Several studies on *P. quinquefolium* extracts, obtained from field-cultivated plants, can be found in the professional literature [36,37,38]. Yet, there are hardly any comparative studies on antimicrobial properties of *P. quinquefolium* plant material obtained with the application of traditional and biotechnological methods. Antimicrobial analysis of this study showed that all tested extracts exhibited activity against both Gram-positive and Gram-negative bacteria, but their efficacy was different depending on the strain and type of the extract. The observed activity against *Staphylococcus aureus* and *Staphylococcus epidermidis*, along with relatively lower MIC values for *Escherichia coli*, suggest that ginseng-derived compounds can interact with various bacterial cell envelope structures. Previous research revealed that ginseng extracts can form complexes with membrane sterols in microbial cells, resulting in reduced membrane integrity, pore formation, leakage of cellular contents, and, in consequence, leading to cell death [38]. Antimicrobial actions of ginseng constituents can also include reducing microbial motility, disrupting quorum sensing, interfering with biofilm formation, and promoting the breakdown of established biofilms, thereby diminishing microbial virulence [38,39]. Such mechanisms were observed, for example, against *Pseudomonas aeruginosa* [40]. Our findings showed that all extracts exhibited very weak activity against this bacterium. Wu et al. (2011) reported that Asian ginseng extracts containing ginsenosides Rb1 and Rg1 did not influence the growth rate of *P. aeruginosa* [41]. Other research demonstrated moderate antibacterial activity against *P. aeruginosa*. Additionally, ginseng extracts exhibited a concentration-dependent effect on biofilms, which means that at lower concentrations, they increased bacterial adhesion, whereas at higher concentrations (5%), they disrupted 1-day-old biofilms and eradicated mature 6-day-old biofilms. Fluorescence microscopy further confirmed reduced cell viability cells and biofilm structures compared to untreated controls. Subsequent in vivo studies on non-human models supported these findings [42,43].

The extracts tested in this investigation showed relatively low antifungal activity against *Candida* species, as indicated by high MIC and MFC values. This suggests that *P. quinquefolium* extracts have limited fungicidal potential. Our results regarding antimicrobial properties are consistent with other data. Analysis of literature reports confirmed that ginsenosides exhibit antimicrobial activity against both bacteria and fungi; however, antibacterial effects are more extensively documented and often observed at lower concentrations, whereas antifungal activity tends to be more variable and frequently requires higher doses [40,44]. Ginseng extracts tend to inhibit microbial growth rather than kill it and are not considered to be highly effective against microorganisms in comparison to antibiotics. Yet, they do have some potential that could be used in the future to improve currently administered preparations and therapies.

Roots of the studies extracts exhibit antimicrobial properties and might potentially support treatment of human infections. Hence, their biocompatibility with the ISO 10993-5 standard [45] was evaluated using the recommended line of mouse fibroblasts L929, human Hs68 cell line and AGS cell line with reduced amount of MTT tetrazolium salt. The MTT test is based on the reduction of soluble yellow MTT tetrazolium salt to a blue, insoluble MTT formazan product by mitochondrial succinic dehydrogenase [46]. Results of this study demonstrate that ginseng-derived extracts exhibit dose-dependent cytotoxicity against AGS gastric cancer cells and normal fibroblast cell lines (Hs68 and L929). This pattern is consistent with literature reports on the anticancer potential of *Panax ginseng* and its bioactive constituents, particularly ginsenosides. Previous studies showed that ginseng stem/leaf extracts obtained by subcritical water extraction exhibit strong cytotoxicity against AGS cells even at relatively low concentrations. For example, extracts from ginseng leaves and stems demonstrated over 80% inhibition of AGS cell viability at concentrations ranging from 0.25 to 2.5 mg/mL in MTT assays. In contrast, the present study observed moderate cytotoxicity only at higher concentrations (≥2.5 mg/mL), which implies that levels of active compounds in the tested extracts may be lower or the extracts may demonstrate different phytochemical composition. This discrepancy highlights the importance of extraction method, plant material, and metabolic profile, as it has been shown that processing conditions (e.g., subcritical water extraction) or the presence of metabolites such as ginsenoside Rg3, Rh2, Rg5 or Rk3 can significantly enhance the cytotoxic potency of ginseng preparations [47]. This is supported by studies showing that the biological activity of ginseng extracts is closely related to the structure and composition of ginsenosides, with less polar derivatives often displaying stronger anticancer activity [48]. Despite overall agreement with the literature, a notable limitation of the present results is the relatively high concentration required to achieve cytotoxic effects. In many studies, ginseng-derived compounds exhibit cytotoxicity in the µg/mL concentrations, particularly when purified fractions are used. This suggests that the crude extracts tested here may contain a lower proportion of active constituents, or that antagonistic interactions between compounds may reduce overall potency [49].

In addition, earlier research confirms that ginseng extracts exhibit low cytotoxicity toward normal fibroblasts, including Hs68 cells. For instance, treatment with ginseng-derived preparations did not significantly reduce Hs68 cell viability within a broad concentration range and even showed protective effects under stress conditions [50]. Similarly, studies using normal immune or fibroblast-like cells have revealed minimal cytotoxicity (<20%) following treatment with ginseng extracts [47]. These findings strongly support the safety profile observed in the present study, particularly within concentration ranging between 0.5 and 2.5 mg/mL.

Various cytotoxic activity between different extract sources, i.e., field-cultivated roots and hairy root cultures, is another important aspect. Higher cytotoxicity of field-derived extracts toward AGS cells observed in this study may reflect environmental influences on both ginsenoside accumulation and biosynthesis of other metabolites that were not examined in this research. In vitro cultures, such as hairy roots, may exhibit clone-dependent variability in metabolite production, leading to reduced or variable cytotoxic effects.

Finally, it should be noted that the MTT assay evaluates mitochondrial metabolic activity rather than direct cell death. This method is widely accepted. Yet, it may not fully capture the complexity of cytotoxic mechanisms, particularly of compounds that affect cellular metabolism but do not induce immediate cell death. Previous studies on ginseng demonstrated that its anticancer effects are often mediated through apoptosis and epigenetic regulation, including modulation of DNA methylation and gene expression. Therefore, additional assays (e.g., apoptosis markers, ROS generation, modification of signaling pathways, or cell cycle analysis) would enable one to understand the mechanisms underlying the effects observed in this study [51,52,53].

In conclusion, the findings presented here are consistent with current literature reports revealing that ginseng-derived extracts demonstrate moderate, concentration-dependent cytotoxic effects on the tested cell lines. However, relatively low potency observed here in contrast to some published data suggests that further optimization of extraction methods and compound isolation is required. These results make up evidence for the potential of ginseng as an anticancer agent as well as emphasize the importance of standardization and detailed phytochemical characterization.

Monocytes are the most sensitive cells and, upon activation, rapidly induce a cascade of inflammatory responses. Only controlled inflammation leads to revascularization and regeneration of injured tissue. However, strong activation of monocytes by biomaterial components or contaminants, such as endotoxins, may induce acute inflammation, potentially leading to pus formation, tissue degradation, and disruption of cell barriers [54]. THP1-Blue™ cells, like other monocytes, respond to ligands, such as peptides, endotoxins, and glycoconjugates, through toll-like receptors (TLR): TLR-2, TLR-4, TLR-5, TLR-6, and TLR-8. Upon TLR stimulation, NF-κB is activated, and the SEAP enzymatic marker is subsequently secreted into the cell culture milieu. In this study, we show that in the milieu of LPS, THP-1 × Blue monocytes responded with a high level of SEAP, confirming the activation of nuclear transcription factor NF-kappa B. Results obtained in the NF-κB activation assay clearly demonstrate that extracts derived from *Panax quinquefolium* indicate absence of NF-κB activation in THP1-Blue™ monocytes. In contrast to the strong activation observed for the positive control (LPS), all the tested samples maintained SEAP activity at levels comparable to the negative control and consistently below the activation threshold. This indicates that, under the tested conditions, the extracts do not stimulate NF-κB-dependent signaling pathways.

NF-κB is a central regulator of innate immune responses, controlling the expression of numerous pro-inflammatory cytokines and mediators. Its activation is typically associated with recognition of pathogen-associated molecular patterns, such as lipopolysaccharides, via Toll-like receptors. Recent studies emphasize that excessive or prolonged NF-κB activation is linked not only to acute inflammation but also to chronic inflammatory diseases [55,56]. Therefore, the absence of NF-κB induction in the present study suggests that the tested extracts are free from immunostimulatory contaminants and do not activate TLR-mediated pathways. This is particularly important for plant-derived preparations, which are prone to endotoxin contamination.

The lack of any dose-dependent increase in the SEAP activity further supports the conclusion that the extracts are immunologically inert in this model. No NF-κB activation was observed even at the highest concentrations, which implies that bioactive constituents, including ginsenosides, do not exert pro-inflammatory effects on monocytes. Recent scientific reports increasingly highlight anti-inflammatory and immunomodulatory properties of ginsenosides, which are often associated with inhibition of NF-κB signaling, suppression of cytokine production, and modulation of upstream pathways such as MAPK and PI3K/Akt [18,53].

From a biomedical perspective, the absence of NF-κB activation is highly desirable. Uncontrolled activation of monocytes and macrophages can lead to excessive inflammatory responses, resulting in tissue damage and impaired regeneration. Consequently, materials that do not stimulate NF-κB are considered more suitable for therapeutic use, especially in long-term therapies.

A joint analysis of our findings and the previously demonstrated antimicrobial activity and low cytotoxicity toward normal fibroblasts revealed a favorable biological profile of the tested extracts. Although their direct antimicrobial potency is moderate in comparison to the potency of conventional drugs, the lack of pro-inflammatory activity and good biocompatibility reveal that the extracts may be potentially applied as supportive therapeutic agents that do not exacerbate inflammation.

In conclusion, the studied extracts of *P. quinquefolium* demonstrate immunological safety, as it was assessed by NF-κB activation in monocytes. Combined with their bioactivity, properties of the extracts prove their potential application in pharmaceutical and biomedical sciences. However, further studies are required to clarify molecular mechanisms of action of the extracts and to confirm their effects in in vivo models.

## 4. Materials and Methods

### 4.1. Ground and Hairy Root Cultivation

The research material consisted of two types of roots of *P. qunquefolium*—obtained traditionally (from field) and biotechnological methods (cultures of transformed root, also known as hairy root cultures). The field-cultivated roots of American ginseng were obtained from the Agricultural University of Lublin. They were grown for three years on experimental fields on loamy sand cultivated in climatic conditions of the Lublin region (Poland).

The cultures of three clones of *P. quinquefolium* L hairy roots were obtained previously. The hairy root cultures were grown in darkness in 80 mL of B-5 medium without hormone. The cultivation was performed on a rotary shaker (70 rpm) at 26 °C. The cultures were long-term and originated from passages 237–240. Each passage lasted 4 weeks. The inoculum was approximately 300 mg fresh weight (f.w.) and 29.0 mg dry weight (d.w.) of plant material.

### 4.2. Determination of Triterpene Saponin Content

#### 4.2.1. Sample Preparation

Before being used in the study, the field-cultivated roots were dried and powdered, and the hairy root cultures were lyophilized. One gram (g) of plant material was extracted with methanol (50 mL) three times for 30 min. The extracts were collected and evaporated to dryness. Next, the obtained extracts were subjected to a cleaning procedure using an SPE column with octadecyl (C18) [57].

#### 4.2.2. Quantitative Analysis of Ginsenosides with the HPLC Method

Purified extracts were analyzed qualitatively with HPLC method according to [57]. Kochan et al. Briefly, eight ginsenosides, Rb1, Rb2, Rb3, Rc, Rd, Re, Rg1, and Rg2 (all purchased from Sigma Aldrich, Darmstadt, Germany) were determined in the studied plant material. The analysis was performed on an Agilent (Santa Clara, CA, USA) Technology 1200 liquid chromatography apparatus. Ginsenosides were separated on the reverse C18 column. As a mobile phase, a mixture of acetonitrile (A) (J.T. Baker, Deventer, The Netherlands) and water (B) (J.T. Baker, Deventer, The Netherlands) was used in a gradient elution program: 0–16 min: 18% A, 82% B; 17–28 min: 30% A, 70% B; 29–60 min: 32% A, 68% B; 61–64 min: 80% A, 20% B; 65–68 min: 18% A, 82% A. The flow rate was 2 mL/min. Detection of ginsenosides was performed at a wavelength of 203 nm. The level of triterpene saponins (mg/g extract) was determined by comparing the retention time and peak areas between standards and samples.

### 4.3. Biological Properties of P. quinquefolium Extracts

#### 4.3.1. Assessment of Antimicrobial Activity

The antimicrobial test was performed using reference bacterial strains from the American Type Culture Collection (ATCC, Manassas, VA, USA), including Gram-positive strains, *Staphylococcus aureus* ATCC 29213 and *Staphylococcus epidermidis* ATCC 12228; Gram-negative strains, *Escherichia coli* ATCC 25922 and *Pseudomonas aeruginosa* ATCC 27853; and fungal strains, *Candida albicans* ATCC 10231 and *Candida glabrata* ATCC 2001.

Antibacterial and antifungal activities were determined by the broth microdilution according to recommendations of the European Committee on Antimicrobial Susceptibility (EUCAST) as previously described [58]. The antimicrobial activity of the A, B, G hairy root and field root extracts, expressed in mg/mL, was evaluated based on their minimal inhibitory concentrations (MIC) and minimal bactericidal concentrations (MBC) or minimal fungicidal concentrations (MFC).

The lowest compounds concentration resulting in the total growth inhibition was identified as MIC. To determine MBC, 10 μL of culture was collected from each well in which no visible growth of microorganisms was recorded and then plated onto the surface of the culture medium. The cultures were incubated for 24 h at 37 °C and no microbial growth revealed bactericidal activity of the tested compounds. The above-mentioned tests were performed in three independent experiments. Amphotericin B and gentamicin were used as standard antimicrobials. No microbial growth confirmed bactericidal activity of the tested extract. The above-mentioned tests were performed in three independent experiments.

#### 4.3.2. Cell Culture and In Vitro Biocompatibility Assessment

L929 mouse fibroblasts (LGC Standards, Middlesex, UK), human Hs68 skin fibroblasts (CRL-1635™), and human gastric adenocarcinoma cells (AGS) (ATCC^®^ CRL-1739™, Rockville, MD, USA) were cultured at 37 °C in 5% CO_2_ in RPMI-1640 medium with 10% heat-inactivated fetal bovine serum (FBS) and the following antibiotics: penicillin (100 U/mL) and streptomycin (100 µg/mL). Hs68 cells were grown in high-glucose RPMI-1640 containing the same supplements. Cell cultures received a fresh medium two or three times weekly to maintain logarithmic growth. The confluent monolayer was passaged using 0.25% trypsin-EDTA (Biowest, Nuaillé, France).

Cytotoxicity of A, B, and G hairy root, and field root extracts, was tested in L929, Hs68, and AGS cells (2 × 10^5^ cells/mL) according to ISO 10993-5 (2009) and the MTT reduction assay [59]. Extracts were diluted in culture medium at 10, 5, 2.5, 1.25, 0.1, and 0.05 mg/mL. Cells in medium alone served as positive controls, while cells treated with 0.03% H_2_O_2_ served as negative controls (100% cell death). Absorbance was measured at 570 nm using a Multiskan EX plate reader (Thermo Scientific, Waltham, MA, USA). Cell viability was calculated as (absorbance of treated cells/absorbance of control cells) × 100%. All tests were conducted in triplicate.

#### 4.3.3. Monocyte Activation Assay

THP1-Blue™ cells (Invitrogen, San Diego, CA, USA), derived from the human THP-1 monocyte cell line, were used to evaluate whether HA-coated titanium plates could activate monocytes by inducing nuclear factor kappa B (NF-κB). These cells were created by stably integrating an NF-κB-inducible embryonic alkaline phosphatase (SEAP) reporter construct into the THP-1 cell line. NF-κB activation results in SEAP secretion, detectable spectrophotometrically with Quanti-Blue reagent (Invitrogen). THP1-Blue™ cells were cultured at 37 °C in 5% CO_2_ in RPMI-1640 medium supplemented with 10% FBS, 25 mM HEPES, penicillin/streptomycin (100 U/mL), 2 mM glutamine (all from Biowest, Bradenton, FL, USA), and 10 μg·mL blastidin (Invitrogen), as previously described [60]. For the monocyte activation assay, THP-1 Blue™ cells (at 5 × 10^5^ cells/mL) were stimulated with extracts from A, B, and G hairy roots, and field-cultivated root at concentrations of 10, 5, 2.5, 1.25, 0.1, and 0.05 mg/mL. Untreated cells served as a negative control, while cells treated with *Escherichia coli* lipopolysaccharide (LPS) (1 μg/mL) (Sigma-Aldrich) served as a positive control. SEAP activity was measured spectrophotometrically at 650 nm using a Multiskan EX plate reader (Thermo Scientific). All experiments were performed in triplicate.

The IC_50_ was estimated by linear interpolation between the two concentrations surrounding 50% viability. We calculate the classical selectivity index (SI):

SI = IC_50normal_/IC_50 cancer_

where

- IC_50_ (cancer): concentration inhibiting 50% of cancer cell viability;
- IC_50_ (normal): same effect in non-cancerous cells.

### 4.4. Statistical Analysis

All treatment procedures were performed in triplicate by three independent experimenters. The Gaussian distribution of data and residuals was checked with the Shapiro–Wilk test and Q-Q plots. Variances across groups were evaluated using the Brown–Forsythe test data and were analyzed using the Mann–Whitney U test or Kruskal–Wallis test. Any relationships were considered significant for *p* ≤ 0.05. Statistica Version 13.1 software was used for all statistical purposes (STATSoft, Tulsa, OK, USA). Data are presented as mean values ± SD or ±SE.

## 5. Conclusions

*P. quinquefolium* field-cultivated roots and hairy root cultures differed quantitatively and qualitatively in their ginsenoside profiles. The highest total saponin level was determined in clone A of the hairy root cultures.

The tested preparations demonstrated moderate antibacterial activity against *E. coli*, *S. aureus*, and *S. epidermidis*, but were ineffective against *P. aeruginosa* and showed only weak antifungal activity. Among the investigated samples, the field sample exhibited the highest overall biological activity but also the greatest cytotoxicity. In contrast, sample G combined strong antibacterial activity with low cytotoxicity toward mammalian cells, suggesting the most advantageous safety–efficacy profile and making it the most promising candidate for further investigation.

This study is the first to study the influence of hairy root extracts on the AGS (gastric cancer) cell line, Hs68 (human fibroblasts), and L929 (mouse fibroblasts) lines. In addition, for the first time in our research it was stated that tested extracts do not trigger NF-κB activation.

It is also important to point out a limitation of absorbance-based assays, such as the MTT and SEAP assays: the potential interference caused by complex plant extracts, including their intrinsic color, turbidity, or the presence of compounds that may interact with assay reagents and affect absorbance measurements. Although every effort was made to minimize such effects, the possibility of extract-related interference cannot be entirely ruled out. Therefore, the results should be interpreted with this in mind, and future studies using complementary analytical methods may help further validate the observed biological effects.

A limitation of the current study is that the anti-inflammatory potential of the extracts was assessed only by their ability to activate the NF-κB pathway under normal conditions. Although the results suggest that the extracts do not trigger NF-κB activation and thus do not show pro-inflammatory effects in THP1-Blue cells, they do not confirm a direct anti-inflammatory action. Future research should explore the effects of the extracts in models of induced inflammation, such as LPS-stimulated cells, and measure additional inflammatory markers, including pro-inflammatory cytokines (e.g., TNF-α, IL-6, and IL-1β), to more thoroughly evaluate their anti-inflammatory capabilities.

To achieve better effects against cancer cell lines, there is a need to further optimize extraction and isolation procedures of active compounds, as well as to enhance their accumulation through the use of biotechnological methods in the case of transformed roots.

## Figures and Tables

**Table 1:** Classical selectivity index (SI).

Sample	Normal Cell IC_50_ (Hs68)	Cancer Cell IC_50_ (AGS)	SI (Normal/Cancer)	Interpretation
A	~6.0	>10	<0.60	Not selective
B	~8.9	>10	<0.89	Not selective
G	>10	—	N/A	Not determinable
Field	~4.4	—	N/A	No cancer comparison

## Data Availability

The original contributions presented in this study are included in the article. Further inquiries can be directed to the corresponding author(s).

## Supplementary Materials

The following supporting information can be downloaded at: https://www.mdpi.com/article/10.3390/molecules31122117/s1, Table S1: Antimicrobial activity of studied extract, shown as minimal inhibitory concentration (MIC) and minimal bactericidal concentration (MBC) or minimal fungicidal concentrations (MFC). Figure S1: Representative HPLC chromatograms of (A) mixed standards and (B,C) analyzed samples with peak assignments and retention times. Peaks: 1-Rg1 (18.2 min), 2-Re (19.2 min), 3-Rf (28.8 min), 4-Rb1 (30.5 min), 5-Rg2 (31.4 min), 6-Rc (33.2 min), 7-Rb2 (35.2 min), Rb3 (35.2 min), Rd (40.1 min). Table S2: The ginsenoside standard curves.

## Abbreviations

The following abbreviations are used in this manuscript

AGS	human gastric adenocarcinoma cell line
L929	cell line of mouse fibroblasts
Akt	Protein Kinase B
EUCAST	European Committee on Antimicrobial Susceptibility
FBS	fetal bovine serum
HEPES	4-(2-hydroxyethyl)-1-piperazineethanesulfonic acid)—chemical buffering agent
Hs68	cell line of human fibroblasts
ISO 10993-5	international standard for assessing the in vitro cytotoxicity
LPS	lipopolysaccharide
MAPK	Mitogen-Activated Protein Kinase
MBC	Minimum Bactericidal Concentration
MFC	Minimum Fungicidal Concentration
MIC	minimal inhibitory concentrations
MTT	3-(4,5-dimethylthiazol-2-yl)-2,5-diphenyltetrazolium bromide
NF-κB	nuclear factor kappa-B
OA	oleanane
OCT	octillol
PI3K	Phosphoinositide 3-kinase
PPD	protopanaxadiol
PPT	protopanaxatriol
SEAP	embryonic alkaline phosphatase
THP1-Blue™	human monocyte reporter cell line (derived from THP-1)

## References

[1] Potenza M.A., Montagnani M., Santacroce L., Charitos I.A., Bottalico L. (2023). Ancient Herbal Therapy: A Brief History of Panax Ginseng. J. Ginseng Res..

[2] Lee N.H., Jung H.C., Lee S. (2016). Red Ginseng as an Ergogenic Aid: A Systematic Review of Clinical Trials. J. Exerc. Nutr. Biochem..

[3] Xu W., Choi H.-K., Huang L. (2017). State of Panax Ginseng Research: A Global Analysis. Molecules.

[4] Bai X., Qiu Y., Wang J., Dong Y., Zhang T., Jin H. (2024). Panax Quinquefolium Saponins Attenuates Microglia Activation Following Acute Cerebral Ischemia-Reperfusion Injury via Nrf2/miR-103-3p/TANK Pathway. Cell Biol. Int..

[5] Lee H.G., Hur J., Won J.P., Seo H.G. (2024). Ginseng (*Panax ginseng*) Leaf Extract Modulates the Expression of Heme Oxygenase-1 to Attenuate Osteoclast Differentiation. Fitoterapia.

[6] Pan D., Xu L., Chen P., Miao L., Tian Y., Shi D., Guo M. (2024). *Panax Quinquefolium Saponins* Enhances Angiogenesis in Rats with Diabetes and Myocardial Infarction. J. Ethnopharmacol..

[7] Lee C., Lee S., Jang Y.P., Park J. (2024). Anti-Inflammatory Activity of Vacuum Distillate from Panax Ginseng Root on LPS-Induced RAW264.7 Cells. J. Microbiol. Biotechnol..

[8] Gao Q., Li G., Zu Y., Xu Y., Wang C., Xiang D., He W., Shang T., Cheng X., Liu D. (2024). Ginsenoside Rg1 Alleviates ANIT-Induced Cholestatic Liver Injury by Inhibiting Hepatic Inflammation and Oxidative Stress via SIRT1 Activation. J. Ethnopharmacol..

[9] Fan W., Fan L., Wang Z., Mei Y., Liu L., Li L., Yang L., Wang Z. (2024). Rare Ginsenosides: A Unique Perspective of Ginseng Research. J. Adv. Res..

[10] Hou M., Wang R., Zhao S., Wang Z. (2021). Ginsenosides in Panax Genus and Their Biosynthesis. Acta Pharm. Sin. B.

[11] Zhou L., Li Z., Li C., Liang Y., Yang F. (2022). Anticancer Properties and Pharmaceutical Applications of Ginsenoside Compound K: A Review. Chem. Biol. Drug Des..

[12] Jang W.Y., Hwang J.Y., Cho J.Y. (2023). Ginsenosides from Panax Ginseng as Key Modulators of NF-κB Signaling Are Powerful Anti-Inflammatory and Anticancer Agents. Int. J. Mol. Sci..

[13] Xu Y., Bian S., Shang L., Wang X., Bai X., Zhang W. (2024). Phytochemistry, Pharmacological Effects and Mechanism of Action of Volatile Oil from Panax Ginseng C.A.Mey: A Review. Front. Pharmacol..

[14] Li C., Zhou S., Chen S., Xu Z., Xue Q., Wang H., Liu T., Chen W., Feng J., Chen J. (2025). Gut Microbiota-Mediated Immunomodulation Underlies the Anti-Tumor Effects of a Novel Ginseng Polysaccharide. Carbohydr. Polym..

[15] Szymańska A., Nowak A., Lipert A., Kochan E. (2024). Effect of Ginseng Supplementation on Exercise Endurance as a Support for Cardiovascular Disease Management: A Systematic Review and Meta-Analysis. Antioxidants.

[16] Feng L., Liu X., Sun K., Sun Y., Wu W., Chen C., Jin X., Wan X. (2024). Ginsenoside Rb1 Inhibits the Proliferation of Lung Cancer Cells by Inducing the Mitochondrial-Mediated Apoptosis Pathway. Anti-Cancer Agents Med. Chem..

[17] Kang S., Park S.-J., Lee A.-Y., Huang J., Chung H.-Y., Im D.-S. (2018). Ginsenoside Rg_3_ Promotes Inflammation Resolution through M2 Macrophage Polarization. J. Ginseng Res..

[18] Valdés-González J.A., Sánchez M., Moratilla-Rivera I., Iglesias I., Gómez-Serranillos M.P. (2023). Immunomodulatory, Anti-Inflammatory, and Anti-Cancer Properties of Ginseng: A Pharmacological Update. Molecules.

[19] Ma W.-Q., Wang H.-Y., Zhang W.-J., Wang S., Wan X.-F., Kang C.-Z., Guo L.-P. (2021). Effects of ecological factors on shape and ginsenoside of Panax ginseng. Zhongguo Zhong Yao Za Zhi.

[20] Hosakatte N., Kim Y.-S., Jeong C.-S., Kim S.-J., Zhong J.-J., Paek K.Y., Murthy H., Zhong J.J. (2014). Production of Ginsenosides from Adventitious Root Cultures of Panax Ginseng. Production of Biomass and Bioactive Compounds Using Bioreactor Technology.

[21] Ramachandra Rao S., Ravishankar G.A. (2002). Plant Cell Cultures: Chemical Factories of Secondary Metabolites. Biotechnol. Adv..

[22] Zhang B.-Z., Yu H.-Y., Tian C.-F., Yu M., Lee Y.-H., Xiong Z. (2025). Panax Ginseng Root Extract Exhibits Antioxidant and Anti-Inflammatory Properties by Diminishing Oxidative Stress Levels and Modulating the NF-κB Signaling Pathway at Both the Cellular and Tissue Levels in the Skin. Nat. Prod. Commun..

[23] Cho H.T., Kim J.H., Lee J.H., Kim Y.J. (2017). Effects of *Panax Ginseng* Extracts Prepared at Different Steaming Times on Thermogenesis in Rats. J. Ginseng Res..

[24] Shilpa S., Sharma M. (2021). Hairy Root Culture: A Promising Alternative for Enhancing the Production of Biologically Active Compounds. J. Innov. Agric..

[25] Biswas D., Chakraborty A., Mukherjee S., Ghosh B. (2023). Hairy Root Culture: A Potent Method for Improved Secondary Metabolite Production of Solanaceous Plants. Front. Plant Sci..

[26] Liu C., Ahmad N., Tao Y., Hussain H., Chang Y., Umar A.W., Liu X. (2025). Reprogramming Hairy Root Cultures: A Synthetic Biology Framework for Precision Metabolite Biosynthesis. Plants.

[27] Chen W., Balan P., Popovich D.G. (2019). Analysis of Ginsenoside Content (*Panax ginseng*) from Different Regions. Molecules.

[28] Tian Y., Wang Z.-L., Ye L., Wang Y., Di P., Wang H., Chen Y., Zheng D., Yang Y., Xu W. (2025). Comparative Metabolome and Transcriptome Analyses Reveal Mechanisms for Differences in Ginsenoside Profiles of Herbal Medicines Derived from *Panax* Species. Engineering.

[29] Xue Y., Zhang R., Li T., Deng Q., Luo W., Chang R., Zeng D., Tan J., Sun T., Liu Y.-G. (2025). Sustainable Production of Ginsenosides: Advances in Biosynthesis and Metabolic Engineering. Plants.

[30] He S., Gong Y., Deng S., Dou Y., Wang J., Sam H.V., Chen X., He X., Shi R. (2025). Comparative Metabolomic Analysis Reveals Tissue- and Species-Specific Differences in the Abundance of Dammarane-Type Ginsenosides in Three *Panax* Species. Horticulturae.

[31] Titova M.V., Lunkova M.K., Tyurina T.M., Prudnikova O.N., Popova E.V., Klychnikov O.I., Metalnikov P.S., Ikhalaynen Y.A., Vasileva E.N., Rodin I.A. (2024). Suspension Cell Cultures of Panax Vietnamensis as a Biotechnological Source of Ginsenosides: Growth, Cytology, and Ginsenoside Profile Assessment. Front. Plant Sci..

[32] Mohanan P., Yang T.-J., Song Y.H. (2023). Genes and Regulatory Mechanisms for Ginsenoside Biosynthesis. J. Plant Biol..

[33] Aslam B., Wang W., Arshad M.I., Khurshid M., Muzammil S., Rasool M.H., Nisar M.A., Alvi R.F., Aslam M.A., Qamar M.U. (2018). Antibiotic Resistance: A Rundown of a Global Crisis. Infect. Drug Resist..

[34] Rossolini G.M., Arena F., Pecile P., Pollini S. (2014). Update on the Antibiotic Resistance Crisis. Curr. Opin. Pharmacol..

[35] Ynalvez R.A., Compean K. (2014). Antimicrobial Activity of Plant Secondary Metabolites: A Review. Res. J. Med. Plant.

[36] Wang L., Yang X., Yu X., Yao Y., Ren G. (2013). Evaluation of Antibacterial and Anti-Inflammatory Activities of Less Polar Ginsenosides Produced from Polar Ginsenosides by Heat-Transformation. J. Agric. Food Chem..

[37] Sienkiewicz M., Głowacka A., Kowalczyk E., Kochan E. (2015). The Activity of Different Extracts from Panax Quinquefolium L. Cultures against Pathogenic Staphylococcus Aureus with Respect to Ginsenoside Content. Arch. Biol. Sci..

[38] Wang L., Huang Y., Yin G., Wang J., Wang P., Chen Z.-Y., Wang T., Ren G. (2020). Antimicrobial Activities of Asian Ginseng, American Ginseng, and Notoginseng. Phytother. Res..

[39] Kachur K., Suntres Z.E. (2016). The Antimicrobial Properties of Ginseng and Ginseng Extracts. Expert Rev. Anti-Infect. Ther..

[40] Kim Y.-R., Yang C.-S. (2018). Protective Roles of Ginseng against Bacterial Infection. Microb. Cell.

[41] Wu H., Lee B., Yang L., Wang H., Givskov M., Molin S., Høiby N., Song Z. (2011). Effects of Ginseng on Pseudomonas Aeruginosa Motility and Biofilm Formation. FEMS Immunol. Med. Microbiol..

[42] Alipour M., Omri A., Suntres Z.E. (2011). Ginseng Aqueous Extract Attenuates the Production of Virulence Factors, Stimulates Twitching and Adhesion, and Eradicates Biofilms of Pseudomonas Aeruginosa. Can. J. Physiol. Pharmacol..

[43] Alipour M., Omri A., Lui E.M.K., Suntres Z.E. (2013). Co-Administration of Aqueous Ginseng Extract with Tobramycin Stimulates the pro-Inflammatory Response and Promotes the Killing of Pseudomonas Aeruginosa in the Lungs of Infected Rats. Can. J. Physiol. Pharmacol..

[44] Goodwin P.H., Best M.A. (2023). Ginsenosides and Biotic Stress Responses of Ginseng. Plants.

[45] (2009). Biological Evaluation of Medical Devices. Part 5: Tests for In Vitro Cytotoxicity.

[46] Mosmann T. (1983). Rapid Colorimetric Assay for Cellular Growth and Survival: Application to Proliferation and Cytotoxicity Assays. J. Immunol. Methods.

[47] Lee K.A., Kim K.-T., Chang P.-S., Paik H.-D. (2014). In Vitro Cytotoxic Activity of Ginseng Leaf/Stem Extracts Obtained by Subcritical Water Extraction. J. Ginseng Res..

[48] Dong H., Bai L.-P., Wong V.K.W., Zhou H., Wang J.-R., Liu Y., Jiang Z.-H., Liu L. (2011). The in Vitro Structure-Related Anti-Cancer Activity of Ginsenosides and Their Derivatives. Molecules.

[49] Hong H., Baatar D., Hwang S.G. (2021). Anticancer Activities of Ginsenosides, the Main Active Components of Ginseng. Evid.-Based Complement. Altern. Med..

[50] Heo H., Lee H., Yang J., Sung J., Kim Y., Jeong H.S., Lee J. (2021). Protective Activity and Underlying Mechanism of Ginseng Seeds against UVB-Induced Damage in Human Fibroblasts. Antioxidants.

[51] Okuno K., Pratama M.Y., Li J., Tokunaga M., Wang X., Kinugasa Y. (2023). Ajay GoelGinseng Mediates Its Anticancer Activity by Inhibiting the Expression of DNMTs and Reactivating Methylation-Silenced Genes in Colorectal Cancer. Carcinogenesis.

[52] Khan A.Q., Rashid K., AlAmodi A.A., Agha M.V., Akhtar S., Hakeem I., Raza S.S., Uddin S. (2021). Reactive Oxygen Species (ROS) in Cancer Pathogenesis and Therapy: An Update on the Role of ROS in Anticancer Action of Benzophenanthridine Alkaloids. Biomed. Pharmacother..

[53] Hong C.-E., Lyu S.-Y. (2025). Immunomodulatory Activities of Emerging Rare Ginsenosides F1, Rg5, Rk1, Rh1, and Rg2: From Molecular Mechanisms to Therapeutic Applications. Pharmaceuticals.

[54] Mantovani A., Biswas S.K., Galdiero M.R., Sica A., Locati M. (2013). Macrophage Plasticity and Polarization in Tissue Repair and Remodelling. J. Pathol..

[55] Liu T., Zhang L., Joo D., Sun S.-C. (2017). NF-κB Signaling in Inflammation. Signal Transduct. Target. Ther..

[56] Hoffmann A., Cheng G., Baltimore D. (2025). NF-κB: Master Regulator of Cellular Responses in Health and Disease. Immun. Inflamm..

[57] Kochan E., Szymczyk P., Kuźma Ł., Szymańska G., Wajs-Bonikowska A., Bonikowski R., Sienkiewicz M. (2018). The Increase of Triterpene Saponin Production Induced by Trans-Anethole in Hairy Root Cultures of Panax Quinquefolium. Molecules.

[58] Piątczak E., Owczarek A., Lisiecki P., Gonciarz W., Kozłowska W., Szemraj M., Chmiela M., Kiss A.K., Olszewska M.A., Grzegorczyk-Karolak I. (2021). Identification and quantification of phenolic compounds in Salvia cadmica Boiss. And their biological potential. Ind. Crops Prod..

[59] Brzeziński M., Kost B., Gonciarz W., Krupa A., Socka M., Rogala M. (2021). Nanocarriers Based on Block Copolymers of L-Proline and Lactide: The Effect of Core Crosslinking versus Its pH-Sensitivity on Their Cellular Uptake. Eur. Polym. J..

[60] Gonciarz W., Matusiak A., Rudnicka K., Rechciński T., Chałubiński M., Czkwianianc E., Broncel M., Gajewski A., Chmiela M. (2019). Autoantibodies to a Specific Peptide Epitope of Human Hsp60 (ATVLA) with Homology to *Helicobacter pylori* HspB in *H. pylori*-Infected Patients. APMIS.

