# Supplementary material for: Comparison of Ginsenoside Content and In Vitro Biological Activity of Extracts Derived from Hairy Root Cultures and Field-Cultivated Roots of Panax quinquefolium
Source: Molecules. 2026 Jun 16;31(12):2117. doi: 10.3390/molecules31122117 (PMC13306213; doi:10.3390/molecules31122117)
Supplement: Supplementary file 1 [file molecules-31-02117-s001.zip › molecules-4362347-supplementary.pdf]

Table S1 Antimicrobial activity of studied extract, shown as minimal inhibitory concentration (MIC) and minimal bactericidal concentration (MBC) or minimal fungicidal concentrations (MFC)

Microorganism	MIC/MBC/MFC (mg /mL)								MIC=MBC=MFC (mg/mL)	
	A		B		G		korzeń		Gentamicin	Amphotericin B
	MIC	MBC /MFC	MIC	MBC /MFC	MIC	MBC /MFC	MIC	MBC/ MFC		
Gram-negative bacteria										
Pseudomonas aeruginosa ATCC 27853	>10	>10	>10	>10	>10	>10	>10	>10	<0.008	-
Escherichia coli ATCC 25922	2.5	5	1.25	5	1.25	5	1.25	2.5	<0.004	-
Gram-positive bacteria										
Staphylococcus aureus ATCC 29213	5	10	5	10	2.5	10	2.5	10	<0.002	-
Staphylococcus epidermidis ATCC 12228	5	5	2.5	5	2.5	5	2.5	5	<0.002	-
Fungi										
Candida albicans ATTC 10231	10	>10	10	>10	10	>10	10	>10	-	<0.001
Candida glabrata ATCC 2001	10	>10	10	>10	10	>10	10	>10	-	<0.001

Gentamicin and amphotericin B, board-spectrum antibiotics, used as antibacterial and antifungal reference substances, respectively; (-) not tested.

Example chromatograms are given below.

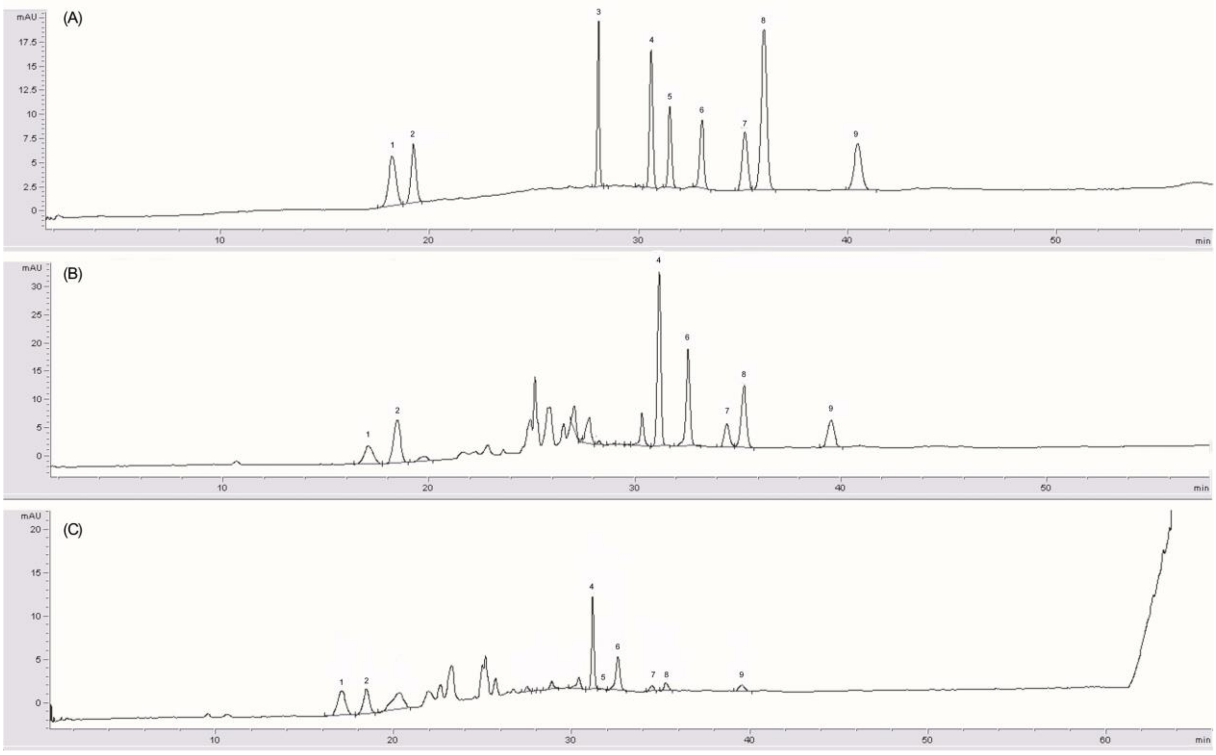

Figure S1 Representative HPLC chromatograms of A) mixed standards and B), C) analyzed samples with peak assignments and retention times. Peaks: 1- Rg1 (18.2 min), 2- Re (19.2 min), 3- Rf (28.8 min), 4- Rb1 (30.5 min), 5- Rg2 (31.4 min), 6- Rc (33.2 min), 7- Rb2 (35.2 min), Rb3 (35.2 min), Rd (40.1 min).

More extensive validation data were provided in an earlier article, which is cited in this version of the manuscript as reference 65. Kochan E, Szymczyk P, Kuźma Ł, Szymańska G, Wajs-Bonikowska A, Bonikowski R, Sienkiewicz M. The Increase of Triterpene Saponin Production Induced by *Trans*-Anethole in Hairy Root Cultures of *Panax quinquefolium*. Molecules. 2018 Oct 17;23(10):2674. doi: 10.3390/molecules23102674 , p. 4.2.2. Quantitative analysis of ginsenosides with the HPLC method). However, we have included the relevant information below and in the Supplementary Material.

Standard solution

Mix ginsenosides: Rb1, Rb2, Rb3, Rc, Rd, Re, Rg1, Rg2, Rf were purchased from Aldrich Sigma, Germany. The concentration of each metabolite was 100µg mL-1. The standard curves were obtained by applying different volumes of ginsenoside mixtures in the HPLC apparatus. The peak area of each ginsenoside standard was recorded. The standard curve was calculated according to the peak area and concentration. In addition, the LOD (limit of detection) and LOQ (limit of the detailed regression equation and validation data) of each standard were recorded.

Table S2. The ginsenoside standard curves

**Ginsenosides**	**Regresion equation**	$\mathbf{r^2}$	**LOD (μg /mL)**	**LOQ (μg /mL)**
Rb1	y=206.23x0.83	0.9992	2.02	6.66
Rb2	y= 149.39x-0.19	0.9988	1.78	5.87
Rb3	y=699.95x-0.55	0.9988	0.26	0.86
Rc	y= 142.57x-0.40	0.9981	1.35	4.46
Rd	y=166.08x-0.46	0.9993	1.80	5.94
Rg1	y=181.38x+0.19	0.9989	0.55	1.82
Rg2	y=181.18x+0.32	0.9989	0.29	0.96
Re	y=155.38x-0.21	0.9989	1.44	4.75
Rf	y=202.63-0.98	0.9998	0.18	0.59
